# It’s Not That Simple: Tobacco Use Identification and Documentation in Acute Care

**DOI:** 10.3390/ijerph10052069

**Published:** 2013-05-21

**Authors:** Patricia M. Smith, Nancy Cobb, Linda Corso

**Affiliations:** 1 Division of Human Sciences, Northern Ontario School of Medicine, Lakehead University, 955 Oliver Road, MS2008, Thunder Bay, ON P7B 5E1, Canada; 2 InfoFinders, 321 Hamstead Court, Waterloo, ON N2K 2B8, Canada; E-Mails: infofinder2@sympatico.ca (N.C.); infofinder2@rogers.com (L.C.)

**Keywords:** tobacco use and dependence guideline, tobacco use identification, tobacco use documentation

## Abstract

This environmental telephone interview scan was designed to identify: (1) how hospitals in one Canadian province incorporated tobacco use identification/documentation systems into practice; and, (2) challenges/issues with tobacco identification/documentation. Participants included 36/139 hospitals previously identified to offer cessation services. Results showed hospitals aided by researchers monitored and tracked tobacco use; those not aligned with researchers did not. The wording of tobacco items most commonly included use within the last 6-months (42%), 30-days (39%), or 7-days (33%), or use without reference to time (e.g., “Do you smoke?”; 39%); wording sometimes depended on admitting form space limitations. The admission process determined where the tobacco item appeared, which differed by hospital—75% included it on an admitting form (75%) and/or nursing assessment (56%); the item sometimes varied by unit. There were also different processes by which the item triggered delivery of cessation interventions; most frequently (69%), staff nurses were triggered to provide an intervention. The findings suggest that adding a tobacco use question to a hospital’s admitting process is potentially not that simple. Deciding on the purpose of the question, when it will be asked and by whom, space allotted on the form, and how it will trigger an intervention are important considerations that can affect the question wording, form/location, systems required, data extraction, and resources.

## 1. Introduction

Screening for tobacco use and dependence during hospitalization is one type of health risk assessment that provides an opportunity to integrate public health measures into clinical environments. Clinical practice guidelines recommend that all hospitals have systems in place to identify and document tobacco use on all patients; doing so will increase the likelihood that clinicians will at least provide a brief cessation intervention, which in turn, could serve to increase tobacco cessation with the large population of smokers who are hospitalized [1]. Although tobacco use identification and documentation in acute care is becoming more prevalent, it is still not a universal standard and compliance is voluntary for hospital accreditation, at least in Canada and the United States [2,3]. For example, of the 165/224 hospitals in the Canadian province of Ontario represented in a provincial survey about tobacco services, 139 reported asking about tobacco use [4] suggesting, generously, that 84% of hospitals ask (139/165); if it is assumed that hospitals that did not respond to the survey do not ask about tobacco, a more conservative estimate would be that only slightly more than half ask (62%; 139/224) and there is a long way to go to achieve 100% compliance with the guidelines.

With the advent of hospitals transitioning to electronic medical records (EMRs), there are a number of examples of how tobacco identification and documentation systems have been integrated into EMRs to prompt clinician interventions [5,6], as well as examples of how the data have been used for public health purposes such as tracking tobacco use prevalence [7] or for billing purposes [8]. A recent Cochrane review showed that adding a tobacco use question to an EMR increases both the expectation that clinicians will intervene with tobacco and as well as clinician actions for treating tobacco use, and when interventions are provided, cessation increases [6] although meta-analyses show the most effective inpatient interventions are those where the intervention is initiated during hospitalization and followed up for at least one month post-discharge [9]. However, integrating tobacco use questions into EMRs does not ensure that cessation interventions will be provided due, at least in part, to the inconsistency of asking and documentation by different healthcare providers [10]. Moreover, if tobacco use data are being collected, challenges include various errors or inconsistencies such as tobacco status occurring in multiple places in a patient’s chart with discrepant information for the same visit, and usability of the data, which in turn, requires standardizing how tobacco use is coded (e.g., check boxes *vs.* open text fields) and determining a standard location in patients’ charts so data extraction programs can be designed [10]. 

To-date, there is also no consensus on the wording for tobacco use questions in acute care. The Center for Disease Control (CDC) [11] was the only reference located that provided a direct recommendation for defining a current smoker—“any smoking in the last month” (either smoking daily or less than daily). This definition is consistent with the US Joint Commission for Accreditation of Healthcare Organizations (JCAHO) [3] criteria for inpatient smoking cessation interventions [12] and Stanford University’s Staying Free inpatient intensive tobacco cessation intervention [13], the most widely-tested hospital-based intervention and the only one to achieve the US Congressionally-Based Top Tier Evidence Standard [14]. The Staying Free definition pre-dated the CDC and JCACHO definitions and was designed for clinical purposes; it was based on ensuring that inpatients who recently quit tobacco and who might be at high risk of relapsing, as well as those who quit tobacco for a planned hospitalization and only had short-term cessation in mind (*i.e.*, to get through the hospitalization), were approached with the opportunity to enroll in a tobacco cessation program that would help them prevent relapse in the long-term [13]. Anecdotally, we have found in our research trials that patients who have quit using tobacco for any amount of time prior to, or during hospitalization (even if for just a day), consider themselves to be non-smokers. This is a drawback to using short time frames to define current smoker as 24 h or not using any time frame (“do you smoke”?).

The current study involved an environmental scan to identify how hospitals in one Canadian province were incorporating tobacco use identification and documentation systems into practice, and to identify challenges and/or issues with adding a tobacco use question. The scan was based on hospitals that had previously responded to a provincial survey about tobacco cessation services and reported that they did ask about tobacco-use [4]. The current study is intended to help move the field forward by elucidating key factors that add complexity to a seemingly simple systems-level change of adding a tobacco use question to the admitting records. The primary outcomes include wording of the tobacco use items being used, the forms on which items appear, and the process by which tobacco use triggers a cessation intervention. Qualitative outcomes include the relevant considerations and challenges surrounding identification and documentation of tobacco use items, and considerations for deciding what indicators hospitals can use to report compliance with tobacco identification and documentation guidelines. 

## 2. Experimental Section

### 2.1. Design and Setting

This cross-sectional telephone-based provincial interview was part of a larger environmental scan to detail the types of cessation services provided in hospitals. The target audience was a sample of the 139 Ontario hospitals that indicated in a recent survey that they offered tobacco cessation services [4]. Selection criteria for inclusion in the current study included: (1) approximately 40 acute care general hospitals that offered inpatient tobacco cessation services; (2) geographical representation from across the province; (3) different sizes/types of hospitals proportional to the Ontario distribution using the categorization defined in the Babayan *et al.* study [4]—small (<100 beds), community (>100 beds), and teaching; and, (4) equal representation from the two turn-key smoking cessation intervention implementation models currently in place in the province—Ottawa Model of Smoking Cessation (OMSC) and the Guideline Implementation Model in NW Ontario (GIM), along with a variety of cessation services that were developed by individual hospitals. Equal representation of implementation models was included to prevent over-weighting the interviews by any one approach. Exclusion criteria included chronic/rehabilitation or specialty hospitals (e.g., mental health). Tobacco cessation coordinators or persons designated the most responsible for coordinating or organizing tobacco cessation services within hospitals were the target recipients of the interview. The interview was based exclusively on publicly accessible information and did not involve personal information of those surveyed and was thus exempted from REB review based on Article 2.2 of the Canadian TriCouncil Ethics Policy [15].

### 2.2. Procedure

A Steering Committee representing various stakeholders—OMSC, Registered Nurses Association of Ontario (RNAO), Smokers Helpline (SHL), Centre for Addiction and Mental Health (CAMH), Cancer Care Ontario (CCO), GIM, and Canadian Cancer Society (CCS)—oversaw the study and provided feedback at various stages. The choice of hospitals to approach began with a review of the following: (1) a list of all 224 Ontario hospitals and a location map; (2) a list of the 165 hospitals that responded to the Babayan *et al.* (2011) survey [4] with a focus on the 139 sites that indicated that they offered tobacco cessation services (information about what individual hospitals offered was not available from the study); (3) recommendations from the Steering Committee based on hospitals they were familiar with; (4) recommendations from others including the Program Training and Consultation Centre (PTCC), the provincial TCAN (Tobacco Control Area Network), and a personal network of colleagues working in hospitals throughout the province; as interviews got underway, recommendations were also made by the hospitals interviewed. Hospitals that were recommended and met the selection criteria were emailed a request for a telephone interview. Interviews were conducted by InfoFinders speaking to the hospital personnel responsible for cessation programs. In some cases information was retrieved from hospital websites. 

### 2.3. Measures

A semi-structured interview designed to be administered over the telephone was developed by expanding a questionnaire previously developed for the GIM hospital initiative. The items relevant to the current study reflected the guideline recommendation systems strategy #1 for hospitals: *have systems to identify and document tobacco use* [1]. To determine what tobacco use questions were being used, the interview item was “What is the question you ask to identify patient tobacco use status?” and included prompts such as: (a) Do you smoke? (b) Have you used any tobacco products in <<time frame>> (time frame included last 30 days, last 7 days, last 6 months)? and, (c) How many cigarettes (or other tobacco products) do you smoke each day on average? Interviewees were also asked “On what form does the tobacco use question appear?” with prompts for: inpatient admissions, emergency department (ED), history and physical/nursing assessment, and other (e.g., tobacco intervention forms). In addition, interviewees were asked to explain the process by which the tobacco use item prompted a tobacco cessation intervention, what clinicians provided the intervention, and whether tobacco use data were collected or analyzed. 

### 2.4. Primary Outcomes

The primary outcomes included wording of the tobacco use items, the form on which the items appeared, and the process by which tobacco use triggered an intervention. Secondary outcomes included summarization and interpretation of interviewees’ comments relevant to considerations and challenges surrounding identification and documentation of tobacco use items and data collection and analyses of tobacco-use. 

### 2.5. Statistical Analyses

Data were entered into FileMakerPro database software for coding open-ended answers and for cleaning, and then entered into SPSS statistical software for analyses. Analyses involved frequency counts and proportions. Where possible, the open-ended comments were coded into discrete categories and analyzed using frequency counts.

## 3. Results and Discussion

### 3.1. Participants

Thirty-six hospitals with equal representation that used the OMSC (n = 12), GIM model (n = 12), and other models (n = 12) were interviewed. Whereas all but one hospital using the GIM model were small, northern, and mostly rural (<100 beds; the exception was a community teaching hospital with >100 beds), the rest of the hospitals using the OMSC or other implementation models were primarily urban—specifically, all but one hospital not using any specific model was large (>100 beds) and all hospitals using the OMSC were large with two thirds representing community hospitals and the rest representing teaching hospitals. 

### 3.2. Tobacco Use Questions

**Figure 1:**
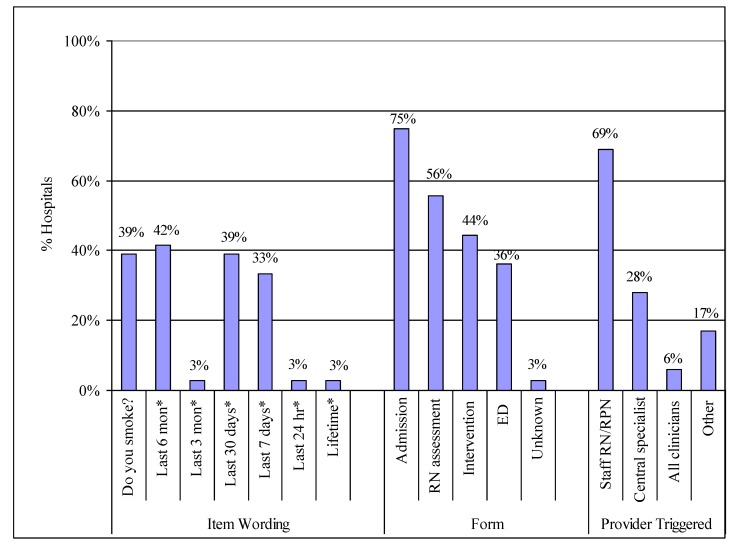
Tobacco use item wording, location, and providers who are triggered to provide a cessation intervention.

Notes: Numbers do not sum to 100% within categories because hospitals sometimes had more than one tobacco use item or had items on more than one form with wording varying by form. As noted in the text, the form name can differ in real or semantic ways so caution should be used when interpreting the “form” findings. Various versions of “Do you smoke?” with no time frame also included “Do you use tobacco products?” and “Tobacco” y/n. * Item wording: Includes some version of the stem question “have you smoked or used tobacco in the last <<time>>?”.

The tobacco-use question varied by hospital in terms of wording, reference to a specific period of time, and the amount of detail. The most commonly asked tobacco questions are in Figure 1. 

Question wording was related to how tobacco use was defined by the implementation model used—6-months and 7-days were used by the OMSC, 30-days by the GIM model, and 7-days among hospitals not using either model (Figure 1). Interviewees also noted that considerations for wording choice included feasibility such as the space available on the admitting form and how the length of the question impacted on the space proposed for other questions on the form. Many hospitals have committees that work with various stakeholders to make decisions about what admitting forms will include and interviewees noted that there is often a lot of competition for form space. For example, some forms might only have room for *“Do you smoke*?*”* or *“Tobacco (yes/no)”* and anything longer, such as a question consistent with the CDC [11] recommendations of 30 days (e.g., *“have you used any tobacco products in the last 30 days”*), would not be approved. Table 1 provides a summary of the wording of tobacco use questions noted by interviewees; due to the range of wording, the varied reasons why the wording was chosen, and the paucity of guidance for wording choice in the literature, suggestions for the possible uses, advantages, and disadvantages for wording choice have been added by the authors as discussion points. Thirty-nine percent of interviewees also reported that their hospitals collect information on amount smoked on the admission record. However, that percentage does not take into consideration the fact that most of the interviewed hospitals provided at least a brief cessation intervention and included amount smoked in the intervention forms.

Although tobacco use in the last 6-months was used by 42% of hospitals, there are no published recommendations in the literature for this definition. Rather, 6-months tends to be a research-related question to assess post-intervention cessation rates rather than define tobacco use prior to an intervention. Interviewees noted that using 6-months to define tobacco use generated a “huge” volume of patients for interventions and that hospitals lacked the resources to continue to screen and provide interventions at that level; in some cases, hospitals that started with 6-months dropped it as a question and reverted to another definition (e.g., 7-days) which interviewees found more clinically-relevant. It is important to consider that defining patients who quit smoking 6-months prior to hospitalization as smokers will serve to artificially inflate the smoking prevalence rates as well as the smoking cessation rates if they remain tobacco-free post-discharge.

**Table 1:** Considerations of different tobacco use questions in research and practice.

Research Uses			Clinical Practice	
Tobacco use	Relative Advantages	Relative Disadvantages	Relative Advantages	Relative Disadvantages
Lifetime	Relevant for lifetime disease risk.	Does not define current users. Not practical for tracking utilization by users *vs.* non-users, tobacco prevalence, or cessation. No published recommendations for this timeframe.	None.	Does not define current smoker. Increases workload of asking patients without it necessarily being relevant to current hospitalization. No published recommendations for this time-frame.
Last 6 months	Comparable to studies/programs using same definition.	Same as lifetime-use. Distorts tobacco prevalence and post-discharge cessation by including patients who have not smoked for >30 days prior to hospitalization. Not relevant for lifetime risk.	None.	Same as life time use. Increases workload by providing interventions to patients who have quit >30 days previous. Distorts post-discharge cessation by including patients who have not smoked for >30 days prior to hospitalization.
Last 3 months	Same as 6-months.	Same as 6-months.	None.	Same as 6-months.
Last 30 days	Comparable to studies/programs using same definition. Relevant current users. Recommended: CDC [11]; accreditation [12]. Relevant for tracking utilization, prevalence, and cessation.	Not relevant for lifetime risk.	Relevant current users and intervention delivery. Captures those who quit for hospitalization or recently quit and at risk for relapse. Recommended: CDC [11]; accreditation [12].	None.
Last 7 days	Comparable to studies/programs using same definition.	Not relevant for lifetime risk. Patients might temporarily quit before being asked so could underestimate prevalence; not optimal for tracking utilization.	Relevant for current users. Captures those who quit ≤7 days who could be at risk of relapse.	Does not capture recent quitters (last 30 days) who might be at risk for relapse or might need medications adjusted due to quitting [16].
Last 24 h	None.	Same as 7 days.	Relevant for current users but not optimal due to disadvantages.	Same as 7 days. Patients might have temporarily quit in hospital before being asked.
Tobacco?/Do you smoke?	Short, fits restricted space on forms.	Same as 7 days.	Same as 24 h.	Same as 24 h.

In hospitals that did not have a centralized admitting system, adding the tobacco use question often involved making changes to many paper forms and/or electronic systems depending on how interconnected the various admitting systems were and whether the admitting forms were standardized across the various units. Interviewees noted that this can be a complex and costly venture and take a long time to achieve due to internal processes such as decision-making about what items to remove from current forms in order to make room for a tobacco use question, obtaining approval for new forms through the Forms Committees, waiting in a queue for the IT department to make changes to electronic forms and develop triggering mechanisms for cessation interventions, and training staff on the new forms and processes. In our own research, we have been working to make changes to the tobacco use item with one hospital for over two years. 

### 3.3. Forms on which Tobacco Use Questions Appear

Answers about on which form the tobacco use item appeared depended on the admission process, which differed by hospital. The different admission processes are summarized in Table 2. Form names also varied greatly across hospitals. In some hospitals “admitting forms” referred to the electronic registration system in a centralized admitting department where the tobacco question was asked by admitting clerks. In other hospitals “admitting form” was synonymous with the “nursing assessment”, regardless of whether this meant the second step in the admitting process where patients were first admitted through a centralized admitting process and then nurses completed an assessment, or whether it was the first step in the admitting process where patients were admitted directly to the unit and there was no central admitting department (see Table 2 for different admitting processes). In some hospitals, multiple forms had to be changed in order to roll out the tobacco use question hospital-wide.

Developing an understanding of the forms that included tobacco use items was further complicated by the various names used for the forms. For example, “admitting forms” were sometimes called “nursing assessment forms”, usually in hospitals that used a decentralized admitting process whereby patients were admitted on the units rather than through a centralized admitting department. In turn, synonyms for “nursing assessment forms”, depending on hospital, included: (1) patient assessment; (2) nursing history; (3) patient history; (4) history and physical (H&P); (5) nursing admission; (6) admitting; (7) admitting history; (8) admitting assessment; (9) admitting questionnaire; and (10) post-admission assessment. In some hospitals, the tobacco question appeared on a separate tobacco intervention form—when it was completed by nurses during a nursing assessment, the form was sometimes not differentiated from the nursing assessment (or one of the synonyms just noted) and it was called the nursing assessment even though it was a separate form, whereas in other hospitals it was differentiated from the nursing assessment and called a tobacco intervention or consult form. 

Taking these interpretations of forms into account, the form where tobacco use was documented varied across hospitals and by the tobacco intervention implementation model they had used, but was usually included in a centralized admitting form or a nursing assessment (Figure 1). Some hospitals reported multiple locations, such as those using the GIM model, all of which had a standardized tobacco use question on 3 standardized forms in 3 different locations (centralized admitting, tobacco cessation intervention form, and ED) and most also had their own idiosyncratic question on the nursing assessment that pre-dated the roll-out of a formalized tobacco cessation program. 

Although all interviewees indicated that they had at least one question in place to ask patients about tobacco use on admission, there was one conglomerate of hospitals where clinicians could choose *not* to ask patients about tobacco use based on their unions and collective agreements. In another hospital, one interviewee noted that *“It’s the expectation that everyone completes tobacco use documentation. We have EMR and you can’t close it out until they have completed all the charting”*, whereas others noted that while the smoking status question is on various forms, they were unable to comment on whether the form was completed consistently as data were not collected on compliance. 

**Table 2:** Varying hospital admitting processes.

Admitting Process	Description
Centralized	A centralized admitting department whereby admitting clerks admit/register all patients and ask a tobacco use question as part of the process.
Decentralized	A decentralized process in which patients are admitted on the units and asked a tobacco use question by nurses during the nursing assessment.
Unit transfers	Unit transfers (used for internal tracking and accounting purposes) involve discharging patients from one unit and re-admitting them to another unit for a different level of care—regardless of whether the hospital uses a centralized or decentralized admitting process, the original admitting information such as tobacco use status does not necessarily follow patients’ records on unit transfers or if patients are not asked about tobacco use on the first unit, there is no record of their tobacco use status for the “transferred” admission and it is usually not asked on the second unit because the “admitting” process is technically completed with the first admission.
Emergency department transfers	Patients are sometimes admitted through the emergency department (ED), which can be similar to a unit transfer but not necessarily especially in small rural hospitals where the ED is often used for general admitting. Also, many community hospitals use holding areas near ED for admission with the current bed shortages and bed-blocking.
Hybrid	There are a number of versions of hybrid processes: In one hospital, most patients were admitted centrally and the tobacco question asked by admitting clerks except in one unit (labour and delivery (L&D)) which had their own admission system with a tobacco use item asked by the L&D nurses; this system was not connected to the main admitting system and L&D admissions were not captured in the centralized database. In small rural hospitals with centralized admitting departments, admitting clerks are often not on duty nights or weekends so a “short admission paper form” not connected to the centralized electronic admission database is used. The short form has only a few basic questions (no tobacco items) and it is completed by a staff nurse (*vs.* admitting clerks). The full admission is completed by admitting clerks when they are back on duty; however, some patients are discharged or transferred before that happens. In some hospitals with central admitting, physicians sometimes call a unit and say they are admitting a patient directly from home to the floor in which case patients arrive without the full registration process being completed by the admitting clerks in centralized admitting.

Many interviewees commented that the tobacco question could get missed; reasons included: (1) the patient was not able to answer during admission (e.g., unconscious, confused, language barriers, medically unstable, *etc.*); (2) in an EMR, the field was not mandatory or could be by-passed so staff did not ask; or, (3) there were methods of admitting that by-passed the standard process. There was general agreement that if the tobacco question was missed on the initial admission (e.g., patient was unconscious or not asked prior to a unit transfer) the question was not asked later. Others noted that the question being asked and documented depended on individual nurses: *“Documentation in a patient record is dependent upon individual nurse initiative and charting fidelity”;* and, *“It (documentation) is inconsistent and dependent upon bedside RN re what to advise and follow-up”*.

### 3.4. Using Tobacco Use Documentation to Trigger Cessation Interventions

The interviews revealed that the process by which the tobacco question triggers an intervention varied by the tobacco cessation implementation model used and whether a centralized or decentralized approach to providing cessation interventions was used. 

A centralized method of intervention delivery involves a specialized tobacco cessation counsellor who either receives a referral request or reviews the daily census to screen for patients who use tobacco and then the counsellor approaches identified smokers at the bedside to offer a cessation program [13]; the centralized method is consistent with randomized trials of intensive interventions (e.g., [16]). 

With a decentralized approach, all clinicians intervene with their own patients [13]; this is consistent with the guideline approach to brief interventions [1] and rarely involves intensive interventions. In some hospitals, documenting tobacco use status and prompting an intervention was a one-step process, in others it was a 2-step process; for the most part, the process was dependent on the implementation model used (Table 3). 

As shown in Figure 1, in the majority of hospitals, staff nurses are triggered by a tobacco use question in their nursing assessment to provide an intervention (decentralized approach); in these cases, a brief intervention was provided. In some hospitals, a centralized tobacco cessation specialist is triggered by the tobacco item to provide the intervention; this can involve either a brief or intensive intervention and the trigger is either a referral from a staff nurse or the review of the daily census. For the two hospitals in Figure 1 that noted “all clinicians” are triggered to provide an intervention, the interviewees noted that a smoking history was embedded in standard of care on every chart and all clinicians can access and document on the standard of care screen at any time during the hospital stay. “Other” in Figure 1 included comments such as: *“cessation interventions are informal and are up to the individual nurses to provide after they ask the tobacco use question”; “there are no systems to trigger an intervention after the tobacco use question is asked”*; and, *“after staff nurses ask the tobacco use question, they can call a specialty unit (radiation or occupational therapist for cancer patients in one hospital and nurses in the cardiac care program in another hospital) to provide interventions to inpatients”*. 

The factors to consider for determining what is needed to trigger an intervention include: (a) the admitting process (Table 1) and whether there is a direct link from the admitting process to trigger an intervention; (b) whether patient registration (admitting) is electronic and has the capacity to trigger an electronic intervention or whether it is paper-based and a paper form needs to be added to charts as part of the admitting process; (c) whether patient charting (after the initial registration process) is electronic or paper; (d) whether all clinicians (or those in an identified profession such as nurses) provide interventions to their own patients (decentralized approach) or whether a designated counsellor(s) provides interventions to all patients across the hospital (centralized approach); and (e) whether a hybrid approach to cessation interventions is taken whereby, for example, all patients receive a brief 5A intervention as defined by the guidelines [1] by staff nurses and some patients also receive an intensive intervention over and above the brief from a cessation specialist. 

**Table 3:** Process by which a smoking cessation intervention is triggered by the tobacco use question.

Approach to Cessation Interventions	Options for Triggering an Intervention by Asking About Tobacco Use
Centralized Approach	Tobacco use identified in the central admitting department and: A cessation specialist prints the daily census, identifies smokers & provides intensive intervention, or Patient names are placed on the smoking census report by the admitting department and faxed to a cessation specialist who provides a brief bedside intervention.
Decentralized Approach	Tobacco use identified in the central admitting but does not trigger an intervention; another tobacco question asked by staff RNs on the H&P triggers an electronic drop-down intervention (or a paper version is attached to the chart) and RNs provide a brief intervention. In decentralized admitting, staff RNs identify tobacco use on nursing assessment/admission; tobacco use activates an electronic RN care plan for smoking cessation. In some hospitals, there is no formal cessation program—interventions are up to individual RNs. All clinicians can access and document in the tobacco use record on the standard care screen; brief intervention is offered if patient interested.
Mixed Decentralized & Centralized Approaches	Options for triggering an intervention: Same as option 1 or 2 above in the decentralized approach plus at patient or clinician request, an informal intensive intervention provided by cessation specialist (social worker, pharmacist). Staff RNs complete the admission form and ask about tobacco; if a smoker is identified, a requisition is faxed by the nurse to a (centralized) smoking cessation specialist who provides the intervention. Staff RNs identify tobacco on nursing assessment; if patient agrees to NRT and wants cessation support, RNs notify cessation specialist (pharmacist, RT) who provides an intervention. Informal process regardless of admitting process: special unit RNs (cardiac) or pharmacists provide brief interventions to all units when requested by staff nurses and time permits.

**Figure 2:**
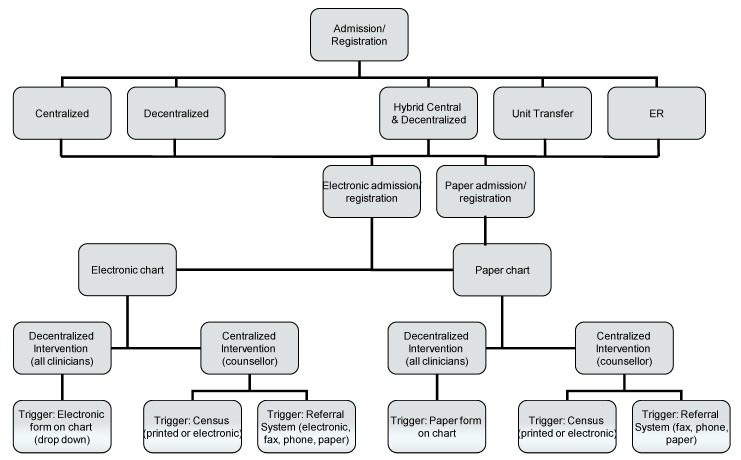
Decision tree to determine the process and forms needed to trigger a tobacco cessation intervention.

Note: Centralized approach to interventions involves a designated counsellor who provides interventions to all patients. Decentralized approach is where all nurses (or other clinicians) are expected to provide interventions to their own patients [10].

The options for using the tobacco question to trigger interventions are summarized in a decision tree in Figure 2. If both admitting and charting are electronic and all nurses, for example, are expected to provide interventions to their own patients (*i.e.*, far left of Figure 2), the tobacco use question can be added to the centralized admitting system and programmed to trigger an intervention form to drop down during a nursing assessment. If the centralized admitting system is not able to trigger an intervention, or charting is paper-based, the tobacco use question must be added to the nursing assessment in order to trigger an intervention; in these cases, whether or not the tobacco use question appears on centralized admitting is irrelevant. However, since a centralized admitting process has a limited number of staff responsible for asking about tobacco, the advantage to including an item on central admitting is that it enhances the likelihood that tobacco use will be asked at least once during hospitalization and the data can be used for purposes such as tobacco use surveillance/prevalence. Regardless of the admission process, if a centralized approach is used for delivering the intervention, designated counsellors must either have access to the daily census to identify patients who use tobacco [13] or there must be a referral system that directs them to specific patients. 

### 3.5. Tobacco Use Identification and Documentation Indicators

The interviewees noted that hospitals aided by researchers (OMSC and GIM) monitored and tracked tobacco use data, but those not aligned with researchers did not. Hospitals, at least in Canada, tend to report programs and services offered rather than quantifying program use and outcomes. That is, it is not common practice to track the prevalence of specific risk factors such as smoking and blood pressures, or co-morbidities such as diabetes except during the roll-out of a new program or because it is mandated by the government. So to meet the minimum standard in the guideline recommendations for tobacco use identification and documentation [1], the indicators most aligned with the general reporting procedures of Canadian hospitals would be to report on the policies and practices in place. For example, the two questions in the Babayan *et al.* report [4] could be used: “Documenting patient smoking status upon admission” and “Standard methodology for the identification of smoking status” (e.g., any tobacco use in the past 30 days?), with answer options: (a) adopted; (b) in the process of adoption; (c) under consideration for adoption within the next two years; or, (d) neither adopted nor under active consideration.

Tobacco use monitoring beyond indicating the hospital’s progress in meeting the minimum standard of identifying and documenting tobacco use would require additional resources. What became clear during the interviews is that as the level of complexity for asking about tobacco use increases, especially given the different methods for admitting and the various forms used, the complexity of tracking patient data also increases. Thus data tracking would need to be justified in relation to the ethos and mandate of hospitals as well as the operating funds required to track the data. 

If tobacco use data are being used for research purposes, the systems set up by researchers cannot always be managed by the hospital, even for electronic data and relatively simple outcomes such as tobacco use prevalence due to the complexity and resources involved, so ongoing data tracking could require additional funding after a research project is over if the level of reporting is to be sustained. Electronic systems using standardized items in standardized locations are the most feasible for monitoring and data extraction [7]. Even then, extraction systems have to be set up through IT departments, which can sometimes be delayed due to waiting lists and could have ongoing additional costs. The data often need to be checked or cleaned depending on how the data will be used, and data summaries and analyses require time, resources, and sometimes special expertise whether done internally or externally (e.g., removing readmissions in very large datasets to maintain independence). 

Since tracking tobacco prevalence can add costs, it is noteworthy that research has shown that it might not be necessary to track actual tobacco use prevalence because prevalence can be reasonably estimated using patient age and the population smoking rates. For example, a study in S. Ontario that screened over 46,000 inpatients [17] and one in NW Ontario that screened over 500,000 ED visits [7] for tobacco use both showed that the tobacco use prevalence among patients mirrored the general population if smoking prevalence was stratified by age. Paradoxically, although data suggest that smokers are more likely to be hospitalized than non-smokers [18], the overall smoking rate for hospitalized inpatients, if not stratified by age, tends to be lower than the general population because adults over the age of 45 years tend to make up the majority of hospitalizations and they have lower smoking rates [13], and the ED rates are likely to be higher than the general population due to a higher proportion of young adults visiting the ED compared to the general population [7]. 

Finally, monitoring and analysis of data from paper-based admitting or charting systems requires resources for personnel to input the information into an electronic format on an ongoing basis, which was an issue identified by some of the hospitals and the component they were likely to drop if the hospitals had limited funding or their special funding for cessation services dried up. The only other option for paper forms is to physically pull charts retrospectively for tracking purposes—not a sustainable task without funding and some would argue not a good use of resources in this time of fiscal restraint. 

## 4. Conclusions

Adding a tobacco use question to a hospital’s admitting process is potentially not as simple as it might first seem. Deciding on the purpose for having a tobacco question on hospital forms, when the question will be asked, who will ask it, and how the question will trigger an intervention are important considerations that can affect the wording of the questions asked, the form-location, the extent of the systems required to support the purpose of asking the question, data extraction, and resources. Other considerations include possible negotiation for space on admitting forms, potentially long wait times for changes due to Forms Committees and IT, and how asking and intervening fit into roles and collective agreements. At a minimum, the question should be asked for clinical reasons [1] in which case the ability to define current users is essential, and a relatively short yet sufficiently long enough time frame should be considered so that patients who quit for a planned hospitalization and are at risk for relapse or those who consider themselves nonsmokers as soon as they are admitted to the hospital are identified. Thus the CDC [11] or JCAHO [3] metric of 30-days is reasonable; the amount smoked could also be included for research purposes or when tobacco use has clinical implications such as possible adjustments to medications that are metabolized differently when patients smoke [19]. Indicators representing compliance with guidelines for identifying/documenting tobacco use [1] could include wording to the effect of having a tobacco identification and documentation system in place; increasing indicators beyond that comes with a cost. 
